# Knowledge, Information, and Data Readiness Levels (KaRLs) for Risk Assessment, Communication, and Governance of Nano‐, New, and Other Advanced Materials

**DOI:** 10.1002/gch2.202200211

**Published:** 2023-05-22

**Authors:** Damjana Drobne, Dmitri Ciornii, Vasile‐Dan Hodoroaba, Nils Bohmer, Sara Novak, Eva Kranjc, Veno Kononenko, Rudolf Reuther

**Affiliations:** ^1^ Department of Biology Biotechnical Faculty University of Ljubljana Večna pot 111 Ljubljana 1000 Slovenia; ^2^ Bundesanstalt für Materialforschung und‐Prüfung (BAM) Division 6.1 Surface Analysis and Interfacial Chemistry Unter den Eichen 87 12205 Berlin Germany; ^3^ Evonik Operations GmbH Rodenbacher Chaussee 4 63457 Hanau‐Wolfgang Germany; ^4^ Environmental Assessments Oberes Lautenbächle 3 77886 Lauf Germany

**Keywords:** cocreation, codesign, knowledge readiness, novel technologies, risk governance, stakeholder engagement

## Abstract

The obvious benefits derived from the increasing use of engineered nano‐, new, and advanced materials and associated products have to be weighed out by a governance process against their possible risks. Differences in risk perception (beliefs about potential harm) among stakeholders, in particular nonscientists, and low transparency of the underlying decision processes can lead to a lack of support and acceptance of nano‐, new, and other advanced material enabled products. To integrate scientific outcomes with stakeholders needs, this work develops a new approach comprising a nine‐level, stepwise categorization and guidance system entitled “Knowledge, Information, and Data Readiness Levels” (KaRLs), analogous to the NASA Technology Readiness Levels. The KaRL system assesses the type, extent, and usability of the available data, information, and knowledge and integrates the participation of relevant and interested stakeholders in a cocreation/codesign process to improve current risk assessment, communication, and governance. The novelty of the new system is to communicate and share all available and relevant elements on material related risks in a user/stakeholder‐friendly, transparent, flexible, and holistic way and so stimulate reflection, awareness, communication, and a deeper understanding that ultimately enables the discursive process that is needed for the sustainable risk governance of new materials.

## Graphical abstract

This paper describes the “Knowledge, Information, and Data Readiness Levels” (KaRL) system for data/knowledge categorization (readiness), which provides a way to align data, information, and knowledge about chemical/material risks with societal needs, values, and requirements. This is accomplished, in part, by integrating all relevant stakeholders at an early stage to propose decisions for risk governance and support safe and sustainable‐by‐design efforts.

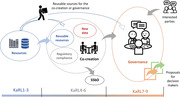

## 1 Introduction

Assessing the benefits and potential risks arising from novel and advanced materials is becoming a more and more complex process due to the multidisciplinarity of the underlying research and the increasing merging of various cutting‐edge technologies, such as nano‐, bio‐, or artificial intelligence technology. In order to properly assess the type and extent of true concerns and compare risks and benefits, the available diversity and great amount of data, information, and knowledge on the material risk must be converted into an actionable and yet easily understandable form. Decisions about risks should be based on a clear risk understanding and a broad and focused interaction between all stakeholders involved, including experts, regulators, industries, and civil societies. It is here where risk governance comes into play, to deal responsibly with uncertain, complex and/or ambiguous risks.

Risk governance can be viewed as the “translation” of scientific expertise, procedural logic, and core principles of governance (e.g., accountability, transparency, effectiveness, efficiency, and strategic vision/focus) into risk‐related decision‐making.^[^ ^30^ ^]^ As described by Renn (2011), “risk governance includes the totality of actors, rules, conventions, processes and mechanisms concerned and how relevant risk information is collected, analyzed, communicated and how management decisions are taken.”^[^ ^31^ ^]^

According to Renn, risk can be classified into four risk classes: simple, complex, high uncertainty, and high ambiguity risks.^[^ ^31^, ^32^ ^]^ But especially complex, uncertain, or ambiguous risks are often characterized by insufficient or low‐quality data, or relevant data not existing at all. So for these types of risks, a new approach to risk assessment is required when compared to simple risks.^[^ ^33^, ^34^ ^]^ As part of this, a stronger involvement of other stakeholders (nonscientist) is needed to ensure that all concerns are properly addressed through transparent, multiway risk communication along the risk governance process.^[^ ^35^ ^]^

To foster multi‐, inter‐, and transdisciplinary collaboration between knowledge, information, and data providers and users, a **Responsible Research and Innovation (RRI) framework** has been recently established by the European Commission to align technological innovation with broader social values.^[^ ^36^ ^]^ The RRI aims to engage the public and responsible actors in the science innovation field to produce ethically acceptable, sustainable, and socially desirable research and innovation outcomes.^[^ ^37^, ^38^, ^39^ ^]^

Similarly, the **EU Open Science initiative** of the European Commission also aims to make research data more available through open and global collaboration and closer ties to society.^[^ ^40^ ^]^ This aligns with the requirement of the EU's Open Data policy (see the EU Open Research Data Pilot) to make high quality, trustworthy, and complete data and metadata available for reuse in a timely manner for all relevant stakeholders. In addition, an international standard‐setting instrument on Open Science has been created in the form of the UNESCO Recommendation on Open Science to codify the aims and measures by which scientific knowledge is to be produced and circulated between stakeholder groups.^[^ ^41^ ^]^

Risk governance strategies could benefit from Open Science through easier accessibility of data, information, and knowledge (Open Access), better data harmonization and comparability (Open Data), and stakeholder engagement (Open to Society). As stated by the UNESCO Recommendation on Open Science (CL/4333),^[^ ^42^ ^]^ “By encouraging science to be more connected to societal needs and by promoting equal opportunities for all (scientists, policy‐makers and citizens), Open Science can be a true game changer in bridging the science, technology and innovation gaps between and within countries and fulfilling the human right to science.”^[^ ^43^ ^]^ (Open) data, information, and knowledge are resources for any knowledge‐based risk governance. But to convert these resources into real opportunities and practical achievements, “operational tools” have to be developed. According to Sen's (2004) capability approach, resources and goods (such as data, information, and knowledge) alone are not enough for satisfactory achievement. For the conversion of resources into outcomes (function), the diversity of existing and future needs and contextual circumstances need to be considered when developing “operational tools.”^[^ ^44^ ^]^

To achieve agreement in risk understanding and governance, concerned parties have to be aware of what types of resources (data, information, and knowledge) are available. With new EU policies on data and knowledge sharing and open science in place, data and information overload and fragmentation will become unavoidable if no, or only insufficient, measures are taken to make data and **knowledge available in a differentiated, clearly structured, and transparently processed way**. Particularly in the case of EN, great efforts have been made in the last decade and a lot of funding has been used to generate safety‐relevant data, but for reuse, they have to be organized in a standardized, structured and meaningful way. In line with FAIR principles, recent recommendations for the efficient reuse of (nano)material safety data have been provided.^[^ ^16^, ^45^ ^]^ In addition, also the FAIR implementation network (the AdvancedNanoIN, or AdvancedNano Implementation Network) was recently established (GO FAIR: AdvancedNano. Retrieved July 5, 2022, from https://www.go‐fair.org/implementation‐networks/overview/advancednano/) for the successful reuse of (nano)material safety data.^[^ ^46^ ^]^ But to convert these resources (FAIR (nano)material safety data and knowledge) into practical achievements, risk understanding and governance “operational tools” have to be developed where all interested parties could participate. The significance of this work is in providing a way to support the safer handling and use of materials, increase the transparency of technical risk assessment, integrate all relevant stakeholders at an early stage, implement the precautionary principle, and above all, contribute to nano‐, new, and advanced material related risk governance.

## 2 Motivation

The motivation for the work described in this paper was the realization that despite a huge amount of existing data, information, knowledge, and tools suitable for the risk‐related activities of nano‐ and other new advanced materials, stakeholders, such as industry, policy makers, regulators, the public sphere, and academics, need support for their (re)use. So one of the key questions may be how to integrate the available scientific and technical data, information, and knowledge and the high diversity of regulatory and other needs into a new data/knowledge categorization (readiness) system that takes all actors, rules, conventions, processes, and mechanisms associated with risk duly into account.^[^ ^47^ ^]^

## 3 Method Development by Adapting a Design‐by‐Analogy Approach

**Figure 1:**
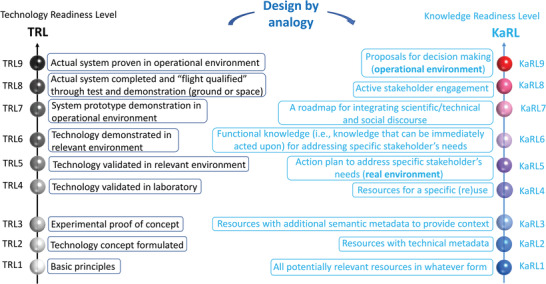
Design‐by‐analogy approach for creating knowledge, information, and data readiness levels (KaRLs) based on the established technology readiness levels (TRLs) and comparison between them.

In our study, a design‐by‐analogy approach has been applied that operates on the premise that a similar solution to a given problem may exist either in an analogous domain or, at least in part, in an analogous solution, and that it can be extracted or elaborated for another problem.^[^ ^48^ ^]^ We have used the technology readiness levels (TRLs) maturity model, as developed at the National Aeronautics and Space Administration (NASA) in the 1970s (**Figure** **1**, Supporting information),^[^ ^49^, ^50^ ^]^ for assessing the status and progress of a “system.” Starting from there, we have elaborated a readiness categorization approach analogous to the basic principle of TRLs that integrates different elements of the nano‐ and other advanced materials risk assessment, communication, and governance process (including data, information, knowledge, tools, participation of interested parties, etc.).

The concept behind maturity/readiness models is not new in the field of nano‐data science. For example, representatives from the collaborating agencies of the Nanotechnology Knowledge Infrastructure (NKI) Signature Initiative (2013)^[^ ^51^ ^]^ elaborated a nomenclature for communicating data readiness. They define readiness as a systematic measure of reliability that is, “The extent to which the provenance of data and their associated metadata are grounded in the methods and procedures of science.” The use of both data readiness levels and metadata qualifiers, as proposed by the NKI Signature Initiative, support an informed sharing of data, augment data citation in print publications, and accelerate the translation of research into practice.^[^ ^51^ ^]^ Furthermore, Lawrence (2017) also elaborated readiness stages for general scientific data to support data analyses as a final goal.^[^ ^52^ ^]^ In his system, the data readiness levels include consideration for how much processing is still required to use particular data, which is relevant in the era of Big Data. The concept of “data readiness levels” has not been further elaborated solely by the nanotechnology community; instead, the domain‐independent FAIR data principles have been adopted for informed data sharing to support data‐driven research.^[^ ^45^, ^53^ ^]^ Similar to data readiness, a concept for knowledge readiness levels (KRLs) was proposed by “RAND Corporation” researchers to estimate the scientific maturity of knowledge products that emerge from health research.^[^ ^54^ ^]^ The aim of these KRLs is to allow evaluations of knowledge products, which include basic research, as well as their application to humans in a real‐world context.

Currently available data maturity and readiness approaches support data analyses or data sharing for data‐driven research or help to evaluate a knowledge product.

**No readiness concept** has yet been developed that aligns scientific and technical components and aspects (data, information, and knowledge) with societal needs, values and requirements.

While assessing the status and progress of technology *development* is the aim of TRLs, the *integration* of scientific data, information, and knowledge with stakeholder needs, and a broad societal involvement is at the core of the KaRLs, as proposed in this paper. In other words, our data readiness levels go beyond assessing tangible products (e.g., scientific results/reports and data reuse). Rather, the **KaRL system assesses the progress and outcome of a *process* that involves multiple stakeholders across various disciplines** to propose decisions for risk governance, or in other cases, to support Safe and Sustainable‐by‐Design (SSbD) efforts (e.g., with the new European Chemicals Strategy for Sustainability [CSS)]).

In both the TRL or KaRL systems, the ultimate aim is to make the process (technology development or knowledge “absorption” by a problem owner[s] and society as a whole) more transparent, easier to understand, and more amenable to clear communication.

## 4 Establishing *K*nowledge, information *a*nd data *R*eadiness *L*evels (*KaRLs*)

To support EN risk assessment, communication, and governance, we have established a nine‐step categorization approach to assess the status and progress of integrating scientific data, information, and knowledge with stakeholder needs and with a broad societal engagement (see **Table** **1** and **Figure** 1). The available resources, i.e., data, information, and knowledge related to nano‐, new, advanced materials are described by KaRLs 1–3. The levels that imply a cocreation process between stakeholders (problem owners) and knowledge providers (experts) are described by KaRLs 4–6. Finally, those levels that integrate knowledge and societal involvement are described by KaRLs 7–9. The KaRL system is a tool to deliver proposals for material risk related decisions.

**Table 1:** Detailed description of individual KaRLs and comparison between each KaRL and corresponding TRL.^[^ ^49^ ^]^

**KaRL levels**	Comparison between Knowledge, information, and data readiness levels (KaRLs) and technology readiness levels (TRLs)	**Assignment of individual KaRL levels**
KaRL 1	KaRL 1: **All potentially relevant resources** in whatever form *TRL 1: Basic principles*	All accessible data including primary data, information and knowledge at any level of FAIRness, in whatever form and source, including published sources, grey literature, e‐lab books, text reports, opinions, review papers, considered relevant by any interested party (expert or stakeholders as problem owners) relevant for a certain topic.
KaRL 2	KaRL 2: **Resources with technical metadata** *TRL 2: Technology concept formulated*	Resources (KaRL 1) accompanied by a reference, structural, statistical, bibliographic, technical supporting information. KaRL 2 are KaRL 1 resources accompanied by metadata, for example in accordance with metadata checklists/standards (e.g., MIRIBEL, MIAN etc.).
KaRL 3	KaRL 3: **Resources with additional semantic metadata to provide context** *TRL 3: Experimental proof of concept*	Resources with technical metadata (KaRL 2) completed with additional (semantic) metadata describing their meaning. KaRL 3 are KaRL 2 resources with additional information that clarifies the meaning of the data (i.e., semantic annotation aligned with defined ontologies, schemata, and others).

KaRL 4	KaRL 4: **Resources for a specific (re)use** *TRL 4: Technology validated in laboratory*	KaRL 4 are KaRL 3 resources with metadata and semantic metadata **assessed for reuse by a (re)use‐specific scoring system** (Klimisch scoring, DaNa2.0, nanoCRED, GUIDEnano scoring, etc.) or according to expert opinion to be fit for a specific purpose, such as modeling, regulatory decision making, safety‐by‐design decision making, hazard characterization, exposure/ risk assessment, hazard communication and others.
KaRL 5	KaRL 5: **Action plan (e.g., Risk Map)** to address specific user (stakeholder) needs *TRL 5: Technology validated in relevant environment*	**KaRL 5 is a risk action plan (e.g., Risk Map), i.e**., a plan of activities and steps to address individual stakeholder's (problem owner's) needs regarding risk (safe innovation, SSbD, and similar topics relevant for individual stakeholders); a Risk Map puts KaRL 1 – 4 resources into a “relevant environment” for addressing stakeholders needs.
KaRL 6	KaRL 6: **Functional knowledge** (i.e., knowledge that can be immediately acted upon) **for addressing specific stakeholder needs** *TRL 6: Technology demonstrated in relevant environment*	**KaRL 6 is an outcome of the risk action plan (KaRL 5),** i.e., **knowledge presentation** as a description, statement, and/or value expressing and describing the knowledge in question that pertains to a single stakeholder, enabling action by one stakeholder (functional knowledge).
KaRL 7	KaRL 7: **A roadmap or framework** for full scale integration of knowledge and involvement of stakeholders (a prototype) *TRL 7: System prototype demonstration in operational environment*	**KaRL 7 is a scheme/a roadmap outlining integration of functional knowledge (KaRL 6)** into a social/reflexive multistakeholder discourse (who is involved, what is the problem, why it is a problem, functional knowledge available, what is missing) to address a specific risk related issue.
KaRL 8	KaRL 8: **Active stakeholder engagement** *TRL 8: Actual system completed and “flight qualified” through test and demonstration (ground or space)*	**KaRL 8** represents an **action** rather than a tangible outcome.^[^ ^53^ ^]^ An actual **discursive engagement** among multiple stakeholders on risk governance with the aim to elaborate risk related decisions.
KaRL 9	KaRL 9: **Proposals for decision makers** *TRL 9: Actual system proven in operational environment*	An outcome of discursive engagement as **a written statement/expert opinion to be** proposed to decision makers. This KaRL represents an **actionable document** (possible decisions) which includes the state of the available evidence (including uncertainty), and a record of the implementation of KaRLs 1–8 for tracking purposes.

The first three **KaRL levels (KaRLs 1–3)** include available resources, i.e., data within the context of a (scientific) investigation as raw data, text reports, opinions, and review papers, among other document types. The metadata qualifiers facilitate grouping, findability, accessibility, interoperability and reusability of resources, i.e., their FAIRification. These basic KaRL levels provide the starting point for risk assessment (RA) and life cycle assessment (LCA)^[^ ^55^ ^]^ and support informed data sharing and the translation of research to technical/scientific knowledge.

The intermediate group of **KaRL categories from 4 to 6** is focused on the cocreation/codesign process, involving knowledge providers (i.e., experts) and a single stakeholder (knowledge users; the problem owners), as is seen in the case described below where, for example, a company works with scientists to address a particular question, problem, or concern. The KaRL 4 data are a scientific/technical input into the cocreation process and includes studies from which the knowledge, information, or data have been extracted for **reuse** to address stakeholder needs. **KaRL 5** and **KaRL 6** are related to the process of cocreation/codesign itself (KaRL 5) and to its outcome (KaRL 6) and cannot be reached without the active participation of the concerned actors to ensure that the result is usable and meets the problem owner's needs.^[^ ^56^ ^]^ More specifically, **KaRL 5** is an action plan (i.e., a **Risk Map)** which includes: fit for purpose data and data of acceptable usability (KaRL 4), a plan to extract additional existing data, information, and knowledge (from KaRLs 1–3), and a list of necessary but nonexistent data that needs to be generated with new studies, in alignment with requirements for regulatory compliance, where needed. It could also involve the use of relevant tools, such as those for read‐across and study grouping or multicriteria decision analysis (MCDA) when desired, as have been developed in recent and ongoing EU projects such as H2020 GRACIOUS or H2020 CaLIBRAte and others.^[^ ^57^ ^]^ **KaRL 6** is assigned to knowledge elaborated in the form of an **actionable document** that is ready for use by stakeholders to make decisions on nano‐ and other material safety for managing risks and allows **traceability** of the data (resources) back to the original studies, refers to relevant regulatory requirements, and provides a list of international standards available/used to address a specific topic. KaRL6 could be a short report, a distinct value (e.g., effective concentration, lethal concentration, etc.), a traffic light presentation, video, cartoon, safety data sheet, or any means to allow communication of knowledge within a stakeholder decision making process.

**Figure 2:**
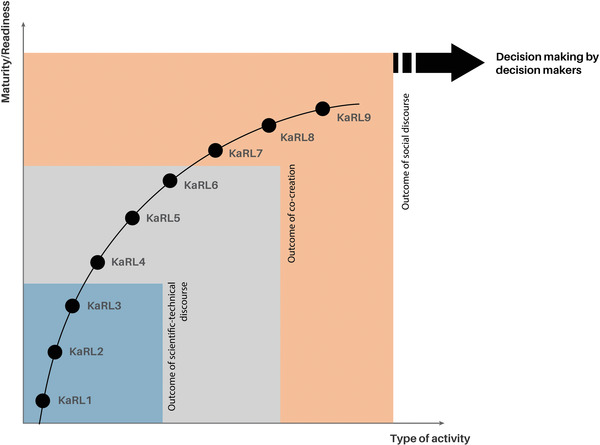
Type of activity to achieve different KaRLs. Lower levels (KaRLs 1–3) are the result of a scientific‐technical discourse, medium levels (KaRLs 4–6) are the outcome of a cocreation process which also incorporates lower KaRLs, and the highest levels (KaRLs 7–9) are based on the discursive engagement of different actors to produce various types of statements (discursive formation) for supporting decision making.

The highest group of **KaRL categories (KaRLs 7 and 8)** is about active discursive engagement between multiple stakeholders, including nonscientists and interested members of the general public. **KaRL 7** is assigned to a **roadmap or framework outlining integration of** the available scientific/technical data, information, and knowledge and the outcomes of cocreation (from KaRLs 1–6) into risk‐related social/reflexive multistakeholder discourse.^[^ ^58^ ^]^ **KaRL 8** is ascribed to the **actual process** of multistakeholder discursive engagement, as already indicated in KaRL 7, which addresses the actual problem, any similar examples and good practices, who is involved, what the problems and conflicts are, what functional knowledge is available, and what is missing. **KaRL 9**, the highest readiness level, is assigned to the outcome of discursive engagement in the form of “a group or family of statements” and reflection from KaRL 8, i.e., discursive formation.^[^ ^59^ ^]^ KaRL 9 is therefore assigned to a group of statements (which may be contradictory or opposing) to support policy, industry, and/or regulatory decision making (see **Figure** **2**). The highest readiness levels can only be reached by integrating functional knowledge with stakeholder participation. An example is described in more detail below.

Figure 1 and Table 1 emphasize the similarities and differences between technology readiness levels (TRLs; developed by NASA) and knowledge readiness levels (KaRLs; developed within the EU NANORIGO project. NANORIGO is an H2020 EU funded project to develop and implement a transparent, transdisciplinary Nanotechnology Risk Governance Framework and Council (www.nanorigo.eu).^[^ ^60^ ^]^ Figure 2 and Table 1 describe the types of activities needed to achieve different KaRL levels.

## 5 KaRL System for Structured and Transparent Integration of Resources and Needs

A key challenging process in the area of risk governance for safe and sustainable novel technologies is the integration of material safety information and value‐focused thinking to channel a critical resource in order to propose better decisions. Here, we present two examples to show how the KaRL system could serve as a framework for the structured and transparent integration of available knowledge (resources), needs and values to support risk decision making.

**Example 1: Using KaRLs to frame the stakeholder (material producer)‐expert interaction**

The intermediate levels of the KaRL system (KaRLs 4–6) involve the integration of both accessible data, information, and knowledge and a cocreation process between stakeholders and experts to address stakeholder‐specific concerns, problems, and/or requirements. The process starts with interaction between an expert and a stakeholder. For example, a material producer (the stakeholder) explains the type of information it needs (e.g., hazardous potential of the new material developed or risk of adverse effects from exposure). In response, the experts search for quality fit for purpose data from available resources (studies, measurements, models) (**KaRLs 1–4, expert involvement**) and review any relevant regulatory requirements. The cocreation process results in **a risk map**, which summarizes the available relevant data, information, and knowledge, including any missing data and regulatory requirements, with the aim of forming a decision (**KaRL 5, cocreation**). The final stage and the main outcome of the cocreation process (**KaRL 6**) is a form of information/knowledge to be used for decision making by the material producer. For example, the outcome of in vitro–in vivo dosimetry modeling data enables the extrapolation of in vitro doses to human exposure levels.^[^ ^61^ ^]^ This information can then guide the decisions regarding additional safety measures by a producer and be used for internal material safety communication.

**Example 2: Using KaRLs to frame multistakeholder engagement**

The highest levels of the KaRL system involve the engagement of a variety of stakeholders and available qualitative and semi‐quantitative information.^[^ ^62^ ^]^ The integration of technical/scientific information and multistakeholder values also lies at the core of multicriteria decision‐making (MCDM) tools^[^ ^62^, ^63^, ^64^ ^]^ that have been used as a vehicle for promoting stakeholder engagement to effectively synthesize and address concerns, preferences, and aspirations from different stakeholder groups.^[^ ^65^ ^]^ MCDM in particular helps to organize information for decision‐making derived from multiple points of view (i.e., disagreement between different decision‐makers or stakeholders) and to assess the tradeoffs between several criteria that are not reducible to only one optimal result. At this knowledge, information and data readiness level (i.e., at **KaRL 7)**, a roadmap for fair and transparent stakeholder engagement is provided that integrates the diversity of views and ideas. It also offers resources (KaRL 4 quality and fit for purpose data) to establish common understanding between disparate stakeholder groups.^[^ ^62^, ^63^ ^]^ When data are missing or highly uncertain, the discursive engagement of different groups is of particular importance (**KaRL 8**) in which experts prepare written statements or expert opinions to be proposed to decision makers along with proposals for decisions (**KaRL 9**).

## 6 Discussion and Conclusions

The KaRL approach introduces a data, information, and knowledge readiness concept to efficiently guide the innovation and risk governance process for engineered nano‐ and related advanced materials. The KaRL roadmap aligns the scientific outcomes (KaRLs 1–3) and stakeholder engagement (KaRLs 4–6) with the formation of interdisciplinary and multistakeholder discourse, as accomplished by using a cocreation process and the discursive engagement of involved stakeholders (KaRLs 7–9). In contrast to TRLs, from which the KaRLs were adapted, the concept we propose guides users in starting either at lower readiness levels, i.e., from scientific data/resources, and proceeding up to higher levels that may include social and multistakeholder debates and discourses on a particular risk, or entering the system at higher levels, starting with public concerns, and subsequently going further down to assure the soundness of the underlying scientific, risk‐based data (such as for hazard or exposure). The ultimate goal in both cases is to arrive at statements or opinions that are based on knowledge and ready to be used for a particular purpose by decision makers (in industry, regulation, or policy) and that also consider the wider societal dimension (values). The KaRL system is a tool that helps to integrate existing scientific knowledge in a structured way with material/product developers, industry, regulators, and the public sphere. The more interaction that occurs between different stakeholders in addressing safe and sustainable materials/products, the more mature the knowledge that is produced for the risk governance of nano‐, new, and advanced materials.

The work we present here was inspired by the strong conviction that “both the “technical and scientific” and the “sociocultural” dimensions of risk need to be equally considered if risk governance is to produce adequate and sustainable decisions and results.”^[^ ^47^ ^]^ The technical and scientific dimension comprises physically measurable outcomes and discusses risk in terms of a combination of potentially positive and negative consequences and their likelihood of occurrence. By contrast, the sociocultural dimension emphasizes how a particular risk is viewed when values, perceptions, and emotions come into play.

According to Ulrich Beck, modern society has become a risk society in the sense that it is increasingly occupied with debating, preventing, and managing risks that have been produced in the process of modernization.^[^ ^66^ ^]^ The awareness of risk is, among others, caused by the awareness of the limits of science, rationality, and knowledge.^[^ ^67^ ^]^ As risk is about the possible consequences of decisions, risk related decisions are of crucial importance.^[^ ^66^ ^]^ This is exactly the type of situation in which the KaRL approach can be used for more transparent communication and complex decision making on nano‐, new, and advanced material related risks that are based on a broad and balanced societal participation as part of the risk governance process.^[^ ^27^ ^]^

The applicability of the KaRL approach as a tool within risk governance to assess data, information, and knowledge readiness and to support final decision making and discursive engagement of all relevant stakeholders is currently being tested within the ongoing H2020 NMBP‐13 project NANORIGO by means of various case studies.

**Concepts on which this work is based**

Michel Foucault's concepts of discourse.^[^ ^59^ ^]^

Amartya Sen's capability approach.^[^ ^44^ ^]^

Ulrich Beck's explanation of risk society and reflexivity.^[^ ^68^ ^]^

**Key Terms Used in This Paper**

*Ambiguous risk*: Ambiguity is usually increased when risks are uncertain and complex, leading to interpretative flexibility.^[^ ^1^ ^]^ Ambiguous risk may be associated with conflicting values and beliefs about consequences,^[^ ^2^ ^]^ incomplete knowledge about probabilities and uncertain events,^[^ ^3^, ^4^ ^]^ or imperfections in human judgment.^[^ ^5^ ^]^

*Advanced materials (AdMa)*: Materials with engineered properties created through the development of specialized processing and synthesis technology, including ceramics, high value‐added metals, electronic materials, composites, polymers, and biomaterials.^[^ ^6^ ^]^ AdMa are understood as materials that are rationally designed to have 1) new or enhanced properties and/or 2) targeted or enhanced structural features.^[^ ^7^ ^]^ In the area of research and development and the corresponding funding systems of the European Commission, advanced materials generally mean materials that have novel or enhanced properties that improve performance over conventional products and processes.

*Cocreation*: An approach used to create value and enhance engagement, collective intelligence, and creativity that involves collaboration between and among experts and stakeholders.^[^ ^8^, ^9^ ^]^

*Codesign*: Participatory/cooperative design; an approach that actively involves different stakeholders to help ensure that the result meets their needs and is usable.^[^ ^10^ ^]^

*Complex risk*: “When risk cascades through a complex system, the danger is not of incremental damage but of “runaway collapse,” or an abrupt transition to a new, suboptimal status quo.”^[^ ^11^ ^]^ Complex risk is related to complex causal (multicausal) relationships and often originates in complexity‐rich political, ecological, social, and economic systems.^[^ ^12^ ^]^

*Data*: The quantities, characters, or symbols making the basis of reasoning or calculation. Data collected in a lab experiment done under controlled conditions is an example of scientific data.

*Data reuse*: Using research data for a research purpose or activity other than that for which it was intended.

*Discourse*: Communication that takes into account the system of thoughts, knowledge, or interaction that constructs the human experience of the world.^[^ ^13^, ^14^ ^]^ It is language shaped by context, including ideas, attitudes, courses of action, beliefs, and practices. Within a risk governance context, discourse is not merely an explanation of scientific data, but an ongoing conversation between involved stakeholders, taking into account the viewpoints and concerns of all.

*Discursive engagement*: Based on Habermas's communicative rationality theory, discursive engagement is a conduit in bringing about authentic social integration and consists of flexible, open‐ended discussion.^[^ ^15^ ^]^

*Discursive formation*: Written and spoken statements with semantic relations and knowledge about something.

*FAIR*: FAIR (findable, accessible, interoperable, reusable) principles put specific emphasis on enhancing the ability of machines to automatically find and use data, in addition to supporting its reuse by individuals (The FAIR Guiding Principles for scientific data management and stewardship).^[^ ^16^ ^]^

*Fit for purpose*: “The main dimension of fitness for purpose, as it is currently used, is the “fit” of the methods to the aim of the research.”^[^ ^17^ ^]^

*Functional knowledge*: Any piece of stored information that can be adapted and applied to different circumstances.^[^ ^18^ ^]^ In the case of the KaRL system, functional knowledge is referred to as acquired knowledge to resolve tasks or to fulfil certain purposes (ready to use).

*Information*: Relevant and timely data (data used within a specific time frame) used to answer questions.^[^ ^10^ ^]^

*Information overload*: Also referred to as information anxiety, infoglut, data smog, analysis paralysis, and information fatigue syndrome.^[^ ^19^ ^]^ Information overload is caused by the sense that a person is losing control over his/her ability to comprehend the meaning of complex and/or large amounts of information.^[^ ^20^ ^]^

*Knowledge*: Information combined with experience to solve problems.^[^ ^10^ ^]^

*Knowledge presentation*: It is considered as a way of expressing the transfer of knowledge between human beings.^[^ ^21^ ^]^

*Risk map*: It is based upon an explicit understanding of users, tasks, and environments. Users are involved throughout the design and development stages. Design is driven and refined by user‐centered evaluations using an iterative process that addresses the whole user experience. The design team incorporates multidisciplinary skills and perspectives.

*Open science*: A movement to make scientific research (including publications, data, physical samples, and software) and its dissemination accessible to all levels of an inquiring society, amateur, or professional. “Open science aims to ensure the free availability and usability of scholar publications, the data that result from scholar research and the methodologies, including code or algorithms, that were used to generate those data.”^[^ ^22^ ^]^

*Operational environment (OE)*: It is derived from the military, including the conditions, factors, and relationships within a given environment that affect an operation, action, or system.^[^ ^23^, ^24^ ^]^ For the KaRL system, this refers to the process of including mature knowledge into the framework of risk governance.

*Primary data*: All available data from different sources, explanations, concepts, and perceptions.

*Prototype*: “…an early sample, model, or release of a product built to test a concept or process.”^[^ ^25^ ^]^ A nearly ready system incorporating all components and processes and proven to be operable.

*Risk governance*: “The term governance describes the multitude of actors and processes that lead to collectively binding decisions. The term risk governance translates the core principles of governance to the context of risk‐related policy making.”^[^ ^1^ ^]^

*Safe and sustainable‐by‐design* (SSbD): It “describes safety measures for the prevention of accidents, illnesses, or environmental damage that are applied during the design stage of a facility, process, practice, material or product.”^[^ ^26^ ^]^

*Simple risk*: The cause for the risk is well known, the potential negative consequences are obvious, the uncertainty is low, and there is hardly any ambiguity with regard to the interpretation of the risk. Simple risks are recurrent and not affected by ongoing or expected major changes. As a consequence, statistics are available and the application of statistics to assess the risks in statistical terms is meaningful.^[^ ^1^ ^]^

*Stakeholder*: A person or group of persons with an interest or concern in something.

*Traceability*: It is “the capability to identify the origins of any data cell within the final analysis table essential for good governance, and almost impossible without a formal system of metadata.”^[^ ^27^ ^]^

*Uncertain risk*: It refers to situations where either the outcomes and/or their probabilities of occurrence are unknown to the decision‐maker.^[^ ^28^ ^]^ More specifically, scientific uncertainty which results in uncertain risk relates to the limitedness or absence of scientific knowledge (data, information) that makes it difficult to precisely assess the probability of undesired effects.^[^ ^1^ ^]^

*Value‐focused thinking*: It puts values at the center of decision‐making. The values for any decision situation are essentially a list of all that we care about achieving in that decision context.^[^ ^29^ ^]^

## Conflict of Interest

The authors declare no conflict of interest.

## Supporting information

Supporting Information

## Data Availability

The data that support the findings of this study are available from the corresponding author upon reasonable request.
