# Supplementary material for: Knowledge, Information, and Data Readiness Levels (KaRLs) for Risk Assessment, Communication, and Governance of Nano‐, New, and Other Advanced Materials
Source: Glob Chall. 2023 May 22;7(7):2200211. doi: 10.1002/gch2.202200211 (PMC10362106; doi:10.1002/gch2.202200211)
Supplement: Supplementary file 1 — Supporting Information [file GCH2-7-2200211-s001.pdf]

Global Challenges
Open Access

Supporting Information

for *Global Challenges.*, DOI 10.1002/gch2.202200211

Knowledge, Information, and Data Readiness Levels (KaRLs) for Risk Assessment, Communication, and Governance of Nano-, New, and Other Advanced Materials

Damjana Drobne, Dmitri Ciornii, Vasile-Dan Hodoroaba, Nils Bohmer, Sara Novak, Eva Kranjc, Veno Kononenko and Rudolf Reuther*

Supporting Information

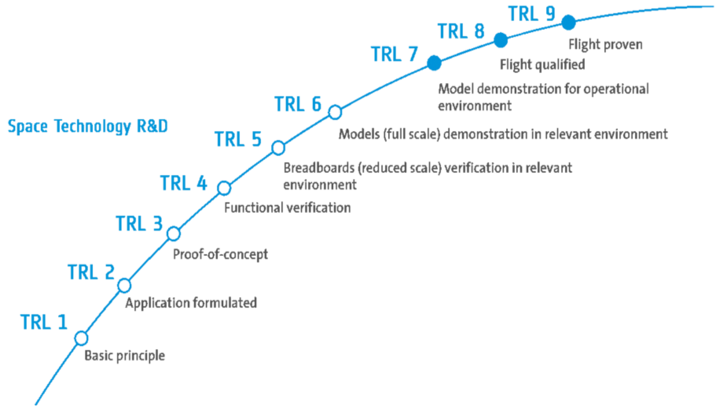

Figure S1. Graphical representation of the 9-level technology readiness level approach (TRL). This approach was developed by NASA for space engineering purposes but can also be translated to other domains.[68]
